# Perspectives on the use of artificial intelligence in Japan: a focus group interview study of healthcare providers

**DOI:** 10.3389/fdgth.2026.1716687

**Published:** 2026-02-11

**Authors:** Atsushi Kogetsu, Kazuto Kato, Beverley Anne Yamamoto

**Affiliations:** 1 Department of Healthcare Ethics, Kyoto University School of Public Health, Kyoto, Japan; 2 Department of Biomedical Ethics and Public Policy, Graduate School of Medicine, The University of Osaka, Suita, Japan; 3 Premium Research Institute for Human Metaverse Medicine (WPI-PRIMe), The University of Osaka, Suita, Japan; 4 North American Center for Academic Initiatives, The University of Osaka, Suita, Japan

**Keywords:** ethical, legal and social implications (ELSI), healthcare provider (HCP), medical artificial intelligence, patient and public involvement (PPI), qualitative research, stakeholder engagement

## Abstract

**Introduction:**

The integration of artificial intelligence (AI) into healthcare is accelerating, raising important questions regarding its implications for clinical practice and the roles of healthcare providers (HCPs). Despite significant technical advances, strategies for engaging frontline stakeholders in developing and implementing medical AI remain underexplored. Therefore, this qualitative study aimed to understand the views and perspectives of stakeholders regarding the use of AI in healthcare.

**Methods:**

We examined the perspectives of 37 healthcare professionals (doctors, nurses, and allied HCPs) in Japan through a series of focus group interviews conducted in 2022. Participants discussed three clinical scenarios involving AI technologies for lung cancer detection, voice recognition during consultations, and healthcare monitoring. A thematic analysis was conducted to explore the views of HCPs on the use of AI in healthcare.

**Results:**

Diverse and sometimes contrasting views were obtained on the benefits, risks, and practical challenges associated with AI in healthcare. Participants reflected on issues such as algorithmic accuracy, bias, responsibility, and the risk of over-reliance. They also raised fundamental questions regarding how AI might redefine the concepts of disease, patient autonomy, and the boundaries between healthcare and everyday life. Although AI was generally considered a support tool for clinical judgement, concerns were raised regarding its potential to reshape healthcare workflows, exacerbate inequalities, and shift professional roles.

**Conclusion:**

The results highlight the importance of involving stakeholders in the early stages of AI development to anticipate its broader implications. Beyond regulating AI as a device, designing healthcare systems that are adaptable, inclusive, and human-centred is necessary. This study provides empirical insight into how HCPs conceptualise AI and emphasises the need for anticipatory governance frameworks grounded in real-world practice.

## 1 Introduction

Artificial intelligence (AI) use in healthcare is rapidly expanding. High expectations for early disease detection, accurate prognosis prediction, and improvements in treatment and efficiency (1, 2) coexist with concerns regarding responsibility, transparency, and social acceptance (3, 4). With the exponential growth of AI applications in healthcare, discussions surrounding the ethical, legal, and social implications (ELSI) are also advancing. Previous debates on AI governance in healthcare have primarily focused on AI as a device (5). For instance, in Japan, efforts are underway to establish a system for approving AI in healthcare as medical devices, with over 20 AI products already approved (6). However, no clear rules govern the use of AI apart from the Ministry of Health, Labour and Welfare's clarification regarding the relationship between AI and Article 17 of the Medical Practitioners' Act, which stipulates that only licensed practitioners are authorised to practice medicine. According to this clarification, the Ministry asserts that practitioners ultimately remain responsible for decisions regarding diagnosis and treatment, even when AI is used in medical practice (7). More recently, the Japan Medical Association published a report entitled “Bioethical Issues in Light of the Accelerating Progress of Medical AI” (8), and academic societies such as the Japanese Society of Radiological Technology, Japanese Society of Pathology, and Japan Primary Care Association issued guidelines (9–11). However, creating a comprehensive framework for AI-based healthcare remains an urgent and critical challenge.

Recent reports and guidelines on AI ethics in healthcare, including those from the World Health Organisation (4, 12), have emphasised the importance of involving stakeholders such as healthcare professionals, patients, and citizens in AI design, implementation, and governance. Nevertheless, research on specific strategies for stakeholder engagement remains insufficient. To address this gap, we conducted the AIDE project (Ensuring the benefits of AI in healthcare for all: Designing a Sustainable Platform for Public and Professional Stakeholder Engagement) as a collaborative research effort between Japan and the UK. The project aimed to clarify strategies for stakeholder involvement and to design an engagement platform (13, 14). Both countries have made substantial investments in medical AI, and various AI applications are currently being developed and implemented (15, 16).

This study presents findings from focus group interviews conducted in Japan as part of this project. An exploratory study was conducted on AI-related perspectives using the Patient and Public Involvement Panel (PPIP) established in Japan and the UK as part of the previous phase of the project (17). Building on this exploratory study, a more detailed investigation was planned. The present study sought to capture the views and perspectives of stakeholders regarding the use of AI in healthcare. Studies on the perceptions and attitudes of stakeholders towards AI have recently increased (18–25). Evidence-mapping and scoping reviews have synthesised international findings on both the perceived positive effects of AI tools on patient care and confidentiality, and the perceived threats they pose to patients' rights and safety from the perspectives of patients, healthcare workers and the general public (26, 27). Related discussions are also emerging in pharmacy practice, where studies have explored pharmacists' perspectives on the use of emerging AI tools and associated practical and ethical concerns (28). In parallel, education of health professionals is increasingly focusing on how AI should be incorporated into curricula and the competencies (i.e., the knowledge, skills, and attitudes) required for clinicians to use AI responsibly (29). However, gathering actual opinions from stakeholders across various contexts is essential for advancing AI development and implementation on a global scale. Accordingly, this study adds the views of stakeholders in Japan, a local context, to the global discourse and presents findings from focus group interviews with healthcare providers (HCPs). This study also sought to identify the expectations and challenges recognised by HCPs regarding AI development and implementation in healthcare, contributing to more effective stakeholder engagement strategies, and ultimately, to the design of AI-based healthcare systems.

## 2 Materials and methods

### 2.1 Study design

We adopted a qualitative descriptive design and conducted online focus group interviews. Scenario-based discussions were employed to elicit participants' perspectives on the use of healthcare AI. The audio-recorded data were transcribed verbatim and analysed using thematic analysis following Braun and Clarke's six-step approach (30). Further details of the analytic procedure are provided in the Data Analysis section (Section 2.5).

We adopted online focus group interviews rather than individual interviews to allow participants to hear and respond to each other's views when discussing the scenarios. This format was expected to facilitate the elaboration of ideas, the articulation of shared understandings within each professional group, and the identification of convergent and divergent perspectives across the professional groups included in the study. Given the small group size (2–4 participants) the discussions were generally collegial rather than strongly contested, and our analysis focused on patterns of meaning in the content of the discussions rather than on detailed interactional dynamics.

### 2.2 Materials

Three written scenarios were developed for use in the focus group interviews. To decide which technological applications to feature in these scenarios, we drew on work conducted within the wider AIDE project. In 2021, the Osaka and Oxford research teams undertook an internal landscape review of AI technologies being developed and/or researched in the University of Osaka affiliated medical hospital and the University of Oxford Trust hospitals. This review took the form of a roundtable discussion in which researchers from both institutions shared cases and challenges related to medical AI development in their clinical settings. Based on this process, we identified four representative clinical applications of AI as potential foci for the scenarios. Draft scenarios were then developed and refined with support from the AIDE Patient and Public Involvement Panels (PPIP) and Expert Advisory Panels in both countries (17). Following further discussion among the two research groups and panels, we decided to focus on three applications.

The final scenarios presented to participants centred on three important emerging clinical applications of AI. One scenario introduced participants to AI-assisted diagnostic imaging systems designed to support lung cancer detection by automatically identifying abnormalities in radiological scans. The second presented natural language processing tools used to generate structured clinical notes from clinicians' verbal or written input, with the aim of reducing the documentation burden. The third scenario described wearable, sensor-based monitoring devices that use AI algorithms to detect irregular heart rhythms and, when necessary, alert the wearer and relevant emergency services. To minimise ordering or association bias, these scenarios were presented to each focus group in a counterbalanced sequence. The full texts of the scenarios are provided in Supplementary File 1.

### 2.3 Participants

Recruitment was performed using purposive sampling (31). We aimed to include diversity across sex, profession (doctors, nurses, and allied HCPs), area of expertise, and care setting (primary or secondary care). During recruitment, we monitored these characteristics and, where feasible, targeted invitations to under-represented groups. HCPs from two large hospitals and clinics in Osaka Prefecture were approached through the research team's network. An electronic flyer describing the study was distributed through these networks, and information about the study was also posted on the AIDE project website. Healthcare professionals who were interested in taking part completed an online entry form created using Google Forms, where they provided basic information and indicated their availability. Focus group sessions were then scheduled by allocating participants to predefined groups (doctors closely involved with AI, doctors not closely involved with AI, nurses, allied HCPs and senior administrative staff) and selecting dates and times that were convenient for each group. In practice, this approach combined purposive sampling with elements of convenience sampling to accommodate participants' schedules (32).

Given that the AIDE project was running in parallel and with a high level of interaction with the Cabinet Office funded AI Hospital project at the University of Osaka, we decided to utilize this access to doctors working on healthcare AI as researchers. We created two categories of doctors for the focus group interviews, those working on healthcare AI development and those who were not. We were not able to replicate this with the other HCP groups as we did not have immediate access to nurses or allied HCPs in sufficient numbers working on healthcare AI projects.

Thirty-seven participants took part in the study: 21 men and 16 women. In terms of age, two were in their 20s, 13 in their 30s, 18 in their 40s, two in their 50s and two in their 60s. The participants included 20 medical doctors (including eight who were closely involved in AI-related research or development), nine nurses and eight allied HCPs (three administrative staff members in management departments, four pharmacists and one radiologic technologist). The inclusion criteria were that participants were aged 18 years or older, currently working in Japan as a doctor, nurse, allied HCP or senior administrative staff member in a hospital or clinic, and able to join an online focus group. Individuals who did not meet these criteria or were unable to participate in an online group interview were excluded. One additional HCP who had initially agreed to participate and completed the entry form was unable to attend the scheduled focus group because of a last-minute scheduling conflict and therefore did not take part in the study.

### 2.4 Data collection

Focus group interviews were conducted online in Japanese in 2022, with 2–4 participants per session, each lasting approximately 90 min. Two of the three scenarios were discussed in each session. Notably, focus group interviews are an effective method for gaining qualitative insights and opinions from people with common attributes, allowing for the expansion of ideas through interactions and the collection of diverse opinions and ideas (33). Scenarios were introduced by the facilitator using a slide deck with illustrations, shared on screen during the online sessions. Discussions were guided by five questions: (1) how participants felt about the AI use, (2) perceived benefits and beneficiaries, (3) concerns about its use, (4) expected changes to their professional role, and (5) who should be involved in decisions about AI development and implementation. The full topic guide is provided in Supplementary File 1. AK and BY served as facilitators for the sessions. Before the start of data collection, the research team reviewed the focus group protocol and topic guide together and agreed on moderation strategies (e.g., using non-leading prompts and encouraging contributions from all participants). Following each session, the facilitators held a brief debriefing to reflect on the facilitation and to note any issues that might have shaped the discussion. Before beginning the interviews, the participants introduced themselves, and the facilitators explained the research purpose, interview procedure, and confidentiality of the discussions. No non-participants were present during the focus group interviews. Each participant took part in a single focus group only; no repeat interviews were conducted. The facilitators took brief field notes during and immediately after each session to record contextual information and initial impressions. Verbatim transcripts were not returned to participants for comment or correction, in order to protect the confidentiality of other participants in each group. The topic guide was not formally pilot-tested.

### 2.5 Data analysis

Audio recordings of the interviews were transcribed verbatim and analysed using thematic analysis (30). The data were inductively analysed, moving from codes to subthemes, themes, and finally higher-order categories. First, initial codes were generated to briefly label the content and meaning of relevant data segments. Similar codes were then collated and grouped to identify common, overarching themes. For some themes, distinct patterns within them were further refined and classified into subthemes. Finally, related themes were organised and consolidated to generate three distinct, higher-order categories that structure the Results section.

This was an iterative process. AK conducted the initial coding and theme development, after which BY reviewed and commented on the emerging coding framework and themes. AK then conducted subsequent rounds of analysis and refinement, with additional reviews and discussions with BY over several months. All coding was conducted using NVivo software (version 2020 R1).

During the course of the study, we discussed the adequacy of the data and judged that the dataset provided sufficient depth and variation to address the study aims. Member checking of the findings with participants was not undertaken in this study. We did not use methodological triangulation (e.g., combining focus groups with other data collection methods) in this study.

### 2.6 Reflexivity

AK, who conducted the facilitation and primary analysis, is a male researcher specialising in the ethical, legal and social implications and governance of emerging medical technologies, with a background as a physician (MD, PhD). This was his first experience of moderating focus group interviews. BY (PhD), who served as co-facilitator, is a female social scientist and a representative of a rare disease patient organisation, and had previous experience of facilitating focus groups. KK (PhD) is a male researcher in biomedical ethics and public policy who contributed to the overall study design and interpretation of the findings but did not take part in data collection or coding. Some participants had pre-existing professional relationships with members of the research team (for example as colleagues in the same institutions), whereas others met the researchers for the first time at the focus group. At the beginning of each session, the facilitators introduced themselves, explained their roles and interests in healthcare AI, and emphasised that participation was voluntary and would not affect participants' employment or clinical evaluations.

### 2.7 Ethics approval

This study was approved by the University of Osaka Hospital Ethical Review Board (20083(T4)-5). Participants received an explanatory document via email outlining the purpose and procedures of the study. After reviewing the document, informed consent was obtained from the participants by replying to the email to indicate their willingness to participate. No one declined to participate after reading the explanatory information.

## 3 Results

Where relevant, quotation attributions indicate the scenario under discussion (S1–S3). The 19 themes identified covered various topics, including technical aspects such as algorithm accuracy and bias; the impacts of AI on healthcare and society; and the processes of AI development, implementation, and usage. These were classified into three categories as follows: “*Perceptions of AI Technology”*, “*Expected Impacts of AI Use”*, and “*Development, Implementation, and Use of AI”* (Table 1). The following sections provide detailed explanations of each category. Notably, participants also devoted considerable attention to broader conceptual and societal implications of AI in healthcare, including potential changes to the concepts of “disease” and “patient” and the boundary between healthcare and everyday life.

**Table 1:** Summary of the results.

Category 1: Perceptions of AI technology
Ensuring the accuracy and validity of AI Data bias Black-box issue Limitations of AI Framing discussions on AI
Category 2: Expected impacts of AI use
Fundamental impacts on existing medicine, medical practices, and healthcare systems Direct impact on healthcare quality and healthcare systems Impact on healthcare professionals’ duties and roles Equitability in healthcare and the expansion of healthcare disparities Concerns about over-reliance on AI Changes in communication between patients and HCPs Impact on the daily lives of patients and citizens Broader societal impacts beyond healthcare
Category 3: Development, implementation, and use of AI
Considerations for AI use in healthcare settings Practical challenges in AI development and implementation The need for education and literacy development How to advance the development, social implementation, and integration of AI into healthcare institutions Benefits and rights associated with the development and use of AI Possible applications and expectations

HCPs, healthcare providers; AI, artificial intelligence.

### 3.1 Category (1): “perceptions of AI technology”

This category includes themes primarily related to AI algorithms – namely “*Ensuring the accuracy and validity of AI”*, “*Data bias”*, the*“Black-box issue”*, and “*Limitations of AI”*. Regarding “*Ensuring the accuracy and validity of AI”*, discussions centred on the need to improve accuracy as a condition for the trustworthiness of algorithms, as well as concerns about fine-tuning, and the need for caution. Specifically, several participants expressed concerns about the difficulty and time-consuming nature of verifying accuracy.

It would make sense if AI could detect lung cancer straight away on something like a computed tomography scan; however, if the goal is to detect the cancer at an even earlier stage, we would still have to wait until the disease actually becomes apparent. (Focus Group 3, doctor-1; Scenario 1)

“*Data bias”* included a range of opinions about biases in training data. While some participants raised concerns about the potential impact of such biases, others argued it was not a significant issue. The “*Black-box issue”* refers to concerns about the inability to understand the rationale behind AI decisions. One participant argued that the reasoning behind an AI's decisions should be made understandable to humans. A prominent opinion in this category concerned the “*Limitations of AI”*, which can be categorised into technological (limitations inherent in the technology) and theoretical (issues that remain unsolvable regardless of technological advancements) limitations. While technological limitations represent challenges that can be addressed with advances in AI technology, theoretical limitations refer to problems that may never be resolved even with technological progress. An example of a theoretical limitation is the inability to verify the accuracy of AI in areas without an established gold standard. Even if the technology was improved, we cannot verify the accuracy without an established gold standard. However, creating a gold standard is challenging such as in the case of super-early detection of lung cancer where the disease cannot be detected without AI. Discussing Scenario 1 (AI-assisted lung cancer detection), one doctor highlighted a practical limitation of ultra-early detection:

AI needs training data, so even systems that learn autonomously still require some form of scoring. In medicine, those scores or “correct answers” are decided by humans, which means they are limited by what humans currently know. There may be correct answers that humans themselves cannot perceive, and in such cases AI would also be unable to make judgments about them. (Focus Group 5, doctor-1; Scenario 1)

This refers to the ability of AI to detect patterns beyond human classification methods and perceptions, which could lead to the creation of new disease concepts by non-human entities, making it a uniquely AI-related issue. Other points raised included “As long as AI is trained based on human judgement, it can never achieve perfect accuracy” and “AI cannot replace entire processes such as diagnosis or creation of medical records”. Many participants viewed AI as a tool to support human judgement, while others envisioned it as something that could “replace humans”. This highlights the difficulty of discussing “AI” as a singular concept, given the varied perceptions among participants.

### 3.2 Category (2): “expected impact of AI use”

This category includes opinions regarding the positive and negative impacts of AI implementation at various levels, including the individual, healthcare institution, and society. “*Fundamental impacts on existing medicine, medical practices, and healthcare systems”* refer to expectations and concerns about how AI implementation could change the nature of medical practices, such as the understanding of existing diseases, diagnosis, and record creation. For example, AI can alter the concept and understanding of diseases and treatment strategies. In relation to Scenario 2 (NLP-based tools to generate structured clinical notes), one participant suggested that AI could also reshape the meaning of medical records by altering whose perspective is retained:

Even with electronic and paper records, doctors pause to think before documenting, so it tends to become a record written from the doctor's perspective. It's hard to say whether that's a benefit or a drawback, but if patients' views become more important in the future, being able to look back on those could be valuable. Right now, what remains is basically the doctor's subjective record; if patients’ perspectives were also recorded, that could be beneficial—although it could also create trade-offs. (Focus Group 4, doctor-1; Scenario 2)

The sub-theme of “*Medicalisation and its impacts”* was created to organise concerns by some participants that the use of AI, for example to detect lesions at a pre-clinical stage, would expand the reach of their practice in ways that may create fiscal as well additional disease burdens. Some participants emphasised that this expansion of healthcare professionals' responsibility could extend to device users (patients and citizens), and that some patients and citizens may either choose not to use AI or may be unable to use it appropriately. Concurrently, concerns were raised regarding the expansion of healthcare professionals' responsibilities through medicalisation.

Contrastingly, the use of wearable devices and AI for medical consultations might enable patients and citizens to rely less on medical institutions, which could be considered as “independence from healthcare” or de-medicalisation.

..When it comes to where AI diagnostics would be used, if it were an environment where the general public could use it, I think patients would be able to understand their illness to some extent without relying on doctors. (Focus Group 5, administration staff-1; Scenario 1)

Under the “*Direct impact on healthcare quality and healthcare systems”* theme, discussions included the prevention of human error, improved accuracy and transparency, use of a wider range of information, and the potential for better communication leading to better healthcare provision. However, concerns were raised about the risks of inappropriate medical interventions, the increased burden on healthcare systems and professionals owing to rising medical demands driven by AI, and the possible increase in healthcare costs owing to unnecessary tests and interventions. In relation to Scenario 1, participants also noted concerns about downstream “cascade” testing and related costs:

One concern is the economic impact of AI on health care. Once sensitivity and specificity improve to a certain level, highly effective AI in medical imaging will emerge. However, to reach that point, many unnecessary tests will have to be conducted. For example, if you are reviewing x-rays and suspect lung cancer, you might order a CT scan, and then discover something else – like an unusual finding in the thyroid or an abnormality in the liver. This would lead to further tests by different specialists, and the medical costs involved could become significant. (Focus Group 5, doctor-2; Scenario 1)

The theme of “*Impact on healthcare professionals’* duties and roles” includes topics related to the workload of healthcare professionals, their roles and expertise, and professional skills. While some participants thought that the introduction of AI would reduce healthcare professionals' burdens, others anticipated that it would lead to new tasks and burdens. One participant pointed out that AI could “prevent them from working at their own pace”, which is related to their sense of professional autonomy and professionalism. Another opinion was that it would be important to “rethink specialisation” with the introduction of AI.

Nurses will need to reconsider what their expertise in nursing is when AI is introduced. I believe this is an important issue to reflect on as AI becomes integrated into nursing practice. (Focus Group 11, nurse-1)

Under the “*Equitability in healthcare and the expansion of healthcare disparities”* theme, some participants expected that AI implementation would reduce differences in the quality of healthcare provided by individual HCPs and that a baseline level of healthcare would be ensured. However, others voiced concerns about unequal access to, and uptake of AI, which could exacerbate existing healthcare inequalities. Specifically, some participants referred to potential gaps between regions and healthcare institutions with differing resources and infrastructure for adopting AI, as well as differences in willingness or ability to use AI among healthcare professionals and patients. “*Concerns about over-reliance on AI”* include healthcare professionals and patients placing excessive trust and dependence on AI. “*Impact on the daily lives of patients and citizens”* refers to concerns about the broader impacts on daily life, such as monitoring or detecting diseases at ultra-early stages, without the involvement of healthcare professionals. Expectations included improvements in health awareness, information visualisation, and peace of mind for families and caregivers, while concerns were also raised, such as “*Being overwhelmed by AI decisions”*, “*Increasing patients’ anxiety and stress”*, “*Discovering diseases at unexpected times”*, and “*Early detection and treatment may not always be the best answer”*. Reflecting on the potential psychosocial impacts of ultra-early detection, one nurse commented:

However, some people may not wish to know. Each individual has their own timing for coming to terms with illness, and learning too early can sometimes cause harm. While early detection and intervention certainly have benefits, the question remains: to what extent can we respect individuals' wishes and values? That is something I continue to be concerned about. (Focus Group 7, nurse-1; Scenario 1)

### 3.3 Category (3): “development, implementation, and Use of AI”

This category covers the challenges, considerations, and expectations regarding the development, implementation, and use of AI. A central topic within this category was “*Considerations for AI use in healthcare settings”*, which includes the explanations and consent procedures for AI, operational rules and systems, intended uses, conditions, positioning, and clinical responsibility. Regarding explanations and consent for AI, opinions on the necessity of informed consent for the use of AI were mixed, with some arguing that it was necessary at first, but would become unnecessary as AI spread throughout society. Some also suggested that disclosing AI usage is important to enable patients to choose their hospitals accordingly. In Japan, patients have the freedom to choose any hospital they wish to visit.

Regarding the results of AI decisions, some participants believed that these should be explained to the patients, while others felt that only the doctor's decisions should be communicated. Operational rules and systems not only concern the rules and systems but also include considerations in the development of AI-enabled devices. In particular, discussions focused on the need to prepare for cases where AI and human judgements diverge or when patients are not happy to have AI used in their diagnostic or treatment pathway. Emphasis was placed on using AI with an awareness of its limitations and the need to build a healthcare system capable of handling the increased demand generated by AI. When reflecting on how AI outputs might be weighed against clinicians' judgement in practice (Scenario 1), one doctor noted:

Here, the issue becomes whether to prioritise AI judgement or the doctor's experience when uncertain. In the case of previous imaging, the doctor's judgement and experience would ultimately take precedence. However, when reasonably good AI becomes available, a time will come when we cannot easily dismiss AI as merely a machine. The ultimate question is whether AI will surpass doctors, and that is the critical issue. (Focus Group 5, doctor-3; Scenario 1)

Regarding the intended use, conditions, and positioning of AI, it was emphasised that the purpose of AI use should be clearly defined, specifying the target population, and using AI when it is clear that it will improve patient prognosis. It was also noted that AI is useful in specific situations or conditions, and should be used as a supplementary tool. Regarding clinical responsibility, broad agreement emerged that doctors should bear the final responsibility, although some argued that doctors should not bear sole responsibility when decisions are made or procedures are followed based on AI. We still have to consider what compensation claims would look like when AI technologies are being used and the relative degrees of responsibility placed on the maker or the doctor.

Furthermore, in situations where a clear decision cannot be reached, the matter becomes somewhat abstract. When AI is used in conjunction with a doctor, the doctor's knowledge and experience may lead to a different diagnosis or treatment approach. This raises concerns for me. For instance, if a misdiagnosis occurs and AI contributes to an incorrect diagnosis or inappropriate treatment, the question arises: who bears responsibility? This is an issue that I find troubling. (Focus Group 11, nurse-2; Scenario 3)

“*Practical challenges in AI development and implementation”* refer to concerns and challenges associated with the development, implementation, and maintenance of AI, including cost and standardisation issues. For example, opinions included “AI development is costly and time-consuming”, “Predicting and verifying cost-effectiveness is difficult”, and “Standardisation across vendors is necessary”. Moreover, “*The need for education and literacy development”* highlights the importance of deepening the understanding of AI and AI-based healthcare for both healthcare professionals and patients/citizens. A particularly important point raised was the need to help people understand not only AI but also the uncertainty inherent in medicine.

Additionally, when it comes to patients' awareness, I find this to be a particularly important topic. After all, if we ask whether medicine is ever absolute, very few would claim that anything can be known with 100% certainty. Of course, in cases of direct observation or when a physical tumour is present, a diagnosis may indeed be definitive. Nevertheless, medicine is fundamentally a matter of probabilities, and I believe the general public also needs to recognize that there are inevitably grey areas in medical practice. (Focus Group 10, doctor-1; Scenario 1)

“*How to advance the development, social implementation, and integration of AI into healthcare institutions”* refers to opinions on who should lead the social implementation of AI and how its integration into healthcare institutions should be managed. Specific stakeholders identified as crucial for involvement include AI vendors and developers; healthcare professionals on the frontlines, such as nurses, general practitioners, and specialists; citizens; patient representatives; advocates for social minorities; public health experts; legal professionals; ethics experts; ethics committees; and private insurance companies, all with a high level of involvement. Simultaneously, the difficulty associated with involving nonexperts in the process was also noted.

With regard to the use of AI, I expect there will be calls for it not to be developed solely by expert groups, but also with the participation of the general public, once they have gained a certain level of understanding. That said, I personally believe it is difficult for the public to be directly involved in the development process. This seems idealistic rather than practical. While citizens may cooperate to some extent, for a major breakthrough such as AI, it is ultimately researchers who should lead the process. What is more important, in my view, is to consider how the results are communicated appropriately to society and how to ensure that AI is applied in ways that are beneficial rather than harmful. I think it is more realistic for the public to be involved at that stage. (Focus Group 5, doctor-3)

Simultaneously, concerns arose that involving key stakeholders in decision-making would hinder progress, with opinions such as “the more people involved, the slower the development and implementation of AI will be”, and that “the government should take the lead in making decisions”.

When it comes to AI, issues such as personal data and the potential for job losses due to AI will arise. In Japan, people are overly sensitive about personal data; therefore, I think the introduction of AI will be difficult. Additionally, since some people will lose their jobs due to AI, professional associations may understandably oppose it. Therefore, if this is going to happen, it would need to be led by the government, or rather, the Ministry of Health, Labour and Welfare, with substantial financial backing and a firm commitment to make it happen. Otherwise, I do not think it will progress. (Focus Group 9, pharmacist-1)

Overall, opinions expressed on this topic combined both macro-level perspectives on the societal implementation of AI and micro-level perspectives on the adoption of AI-based medical devices within individual healthcare institutions.

“*Benefits and rights associated with AI development and use”* refers to opinions regarding the benefits and rights that arise from the development and use of AI. The importance of ensuring that all stakeholders, including those providing training data; patients and citizens involved in AI usage; healthcare professionals; and medical institutions that adopt AI, benefit from its use was emphasised. Concerns were also raised regarding the lack of patient consent for the use of training data and the risks of personal data leakage.

### 3.4 Similarities and differences between the three groups

Across all groups, discussions tended to focus on the second category, “*Expected impact of AI use”*, particularly regarding “*direct impacts on healthcare quality and healthcare systems”* and “*impacts on healthcare professionals’ duties and roles”*. On the topic of how AI might affect healthcare professionals' duties and roles, many participants said AI could not replace their own work because of the depth of expertise involved. However, some felt that other professions could be replaced by AI — which made for an interesting contrast.

Doctors more closely involved with AI placed particular emphasis on “*Considerations for AI use in healthcare settings”* and “*The need for education and literacy”*. For example, while imagining how AI could be used in medical practice, they pointed out that “the workflow in which AI is used is important”. However, doctors not closely involved with AI tended to discuss “*the limitations of AI”*, “*fundamental impacts on existing medicine, medical practices, and healthcare systems”*, and “*concerns about over-reliance on AI”*, with particular focus on questioning the underlying facts.

In the nursing groups, participants highlighted “*impacts on healthcare professionals’ duties and roles”* and “*the potential for AI implementation to either equalise or exacerbate healthcare disparities”*. This theme emerged most clearly in the nursing groups less prominent in the other groups. Both doctors not closely involved with AI and nurses also discussed the “*impact on patients’ and citizens’ lives and lifestyles”*.

Allied HCPs particularly highlighted issues such as “*Ensuring the accuracy and validity of AI”*, “*Data bias”*, and “*Practical challenges in AI development and implementation”*. Notably, discussions about practical challenges emerged most clearly in the allied HCP group; much of it concerned costs, which may reflect the fact that many participants from this group were from hospital management departments.

## 4 Discussion

This study clarified the attitudes and perspectives of healthcare professionals towards AI in the healthcare field. Specifically, based on the scenarios presented, the participants understood the benefits that each AI device could offer while discussing the potential concerns and challenges associated with these technologies.

The development status of medical AI, its regulations, and healthcare systems vary significantly among countries, each facing unique challenges. Japan has made substantial investments in medical AI and is currently establishing regulations to accelerate the development of various medical AI technologies (6, 15). Additionally, Japan has a universal healthcare system, with a strong willingness to use medical technologies such as computed tomography and magnetic resonance imaging (34). Given the ageing and declining population, reducing the burden on healthcare professionals and improving healthcare efficiency are high-priority policy issues (35). These factors contribute to the fact that Japan has a solid foundation for implementing medical AI technologies compared with many other countries.

In addition to the above country-specific contextual factors, our findings should also be interpreted in light of the temporal context of the study. The focus groups were conducted in 2022, a relatively early stage in the deployment of medical AI in Japan. There has been an acceleration in the discourse around and development of healthcare AI since the focus groups were carried out, and contemporary healthcare professionals, including those who participated in this study, are likely to be more aware of and better informed about this area than they were at the time of data collection. However, we contend that the key themes that emerged from the focus group discussions are not transient topics but articulate ongoing concerns and expectations around healthcare AI and allude to challenges and tensions that will be experienced in the field for many years to come.

Recent qualitative studies and reviews have explored how HCPs and other stakeholders view the use of AI in healthcare across a range of settings (18–25). Overall, our findings are broadly in line with this literature: participants anticipated potential benefits such as improved diagnostic accuracy and efficiency, while also raising familiar concerns about algorithmic opacity and bias, medico-legal responsibility, workload, and possible effects on professional roles and the clinician–patient relationship. These concerns were, therefore, largely anticipated, consistent with previous stakeholder studies and our earlier PPIP exploratory study in Japan (17). At the same time, this study adds nuance in several respects. Participants articulated more fundamental questions about how AI may reshape concepts such as “disease”, “the patient”, and the boundary between healthcare and everyday life, which have received less explicit attention in previous HCP-focused work. In addition, by including doctors (both closely involved with AI and not), nurses and allied HCPs, we were able to illustrate how different professional groups foregrounded particular issues – for example, doctors focused on clinical responsibility, the limitations of AI and their implications for core medical concepts, nurses emphasised equity and patients' daily lives, and allied HCPs focused on costs and practical implementation. Finally, by situating these perspectives within the Japanese healthcare system and current policy context for medical AI, the study contributes empirically grounded insights from a setting that remains under-represented in the existing literature.

Recent evidence-mapping and scoping reviews have also synthesised international findings on both the perceived benefits of healthcare AI (e.g., improved care and confidentiality) and perceived threats (e.g., risks to patients' rights and safety) across patients, healthcare workers and the general public (26, 27), suggesting that the concerns and expectations identified in our data resonate with broader global discussions while adding context-specific insights from Japan. As the processes of AI development, implementation, and utilisation progress, fundamental issues arise that transcend cultural and institutional contexts. This section discusses three key points based on the findings of this study.

### 4.1 Fundamental questions raised by the implementation of AI in healthcare

The first issue is that applying AI in healthcare may necessitate reconsidering concepts previously taken for granted in conventional medicine. As this study has revealed, the ability of AI to detect pre-disease stages, well before current definitions of “disease” are established, could lead to an expansion of the concepts of “disease” and “patient”. For example, detecting pre-cancerous lesions through imaging or identifying high-risk conditions for heart and lifestyle-related diseases through health monitoring could spark debates about whether such conditions should be considered “diseases” and whether individuals with these conditions should be called “patients”. This discussion also parallels debates on hereditary diseases in which genetic analysis technologies identify individuals with disease-causing genes in an asymptomatic state (36).

Moreover, the boundary between “medical care provided in hospitals” and “health management in daily life” may become unclear as health monitoring and chatbots improve self-care outside the hospital. This could be seen as “patient independence” in terms of reducing reliance on medical services, but simultaneously as the “medicalisation” of daily life. Consequently, questions arise about how data collected outside hospitals should be handled. Another way to frame this is by asking, “What constitutes “medical care (services)”?” Japan operates a universal health insurance system that covers a wide range of medical services; however, coverage and reimbursement for newly emerging technologies are subject to separate appraisal and fee schedule decisions. As more medical AI technologies are developed, debates are likely to emerge regarding whether, and how, these tools should be reimbursed within the public insurance system. Similarly, discussions will be necessary regarding how medical records should be handled when AI is used, and the role, expertise, and autonomy of healthcare professionals. Taken together, our findings suggest that AI is experienced by HCPs not only as a technical tool but also as a catalyst for renegotiating what counts as appropriate professional judgement, how responsibilities are distributed among doctors, nurses and allied HCPs, and where the boundaries of their clinical work should lie.

Discussion is needed on how AI should be positioned in healthcare (37). Should it be considered “AI” that mimics human intelligence, or “augmented intelligence” that processes complex information beyond human capabilities to promptly support human judgement and decision-making? Is AI a replacement for humans or merely a tool similar to a diagnostic instrument? The answer to these questions will influence the perceptions of the “*limitations of AI”* highlighted by many participants in this study. Many participants positioned AI as a tool to support rather than replace healthcare professionals. However, some expressed doubts about whether AI would continue to be used in this manner once implemented in real healthcare settings.

Therefore, the introduction of AI, a new technology, raises questions about things previously considered self-evident due to their technical impossibility. This issue is common to all emerging technologies, such as life-sustaining treatment (38), brain death organ transplantation (39), genomic analysis technology (36), and assisted reproductive technology (40). In response, identifying what such technologies make possible and which self-evident assumptions they challenge, as well as discussing in advance how these technologies should be implemented, is crucial.

### 4.2 Reflecting stakeholder perspectives at the upstream stage of AI development and use

This study's results suggest the importance of reflecting stakeholder perspectives at the upstream stages of AI development and use. Even if an AI that is useful in one aspect is developed, failing to anticipate its significant impacts on medical practice and citizens' lives during the development phase may prevent the technology from being used as expected in practice. As stated by this study's participants, in the first scenario (AI-based lung cancer detection), even if cancer is detected at an ultra-early stage – which is not yet a treatment target – it could result in a psychological burden for patients. In the second scenario (AI-based voice recognition in consultations), although AI was intended to reduce the burden on healthcare professionals, concerns exist that it could become “difficult to use” in practice without considering its impact on communication between patients and healthcare professionals or how medical records should be handled. Similarly, in the third scenario (AI-based healthcare monitoring), recording daily vital data risks causing patients to become overly anxious – an issue that should be identified and addressed beforehand.

To avoid such issues, having discussions that consider clinical use from an early stage – with active involvement of stakeholders such as patients, citizens, and healthcare professionals – is essential. The importance of stakeholder involvement in medical AI discussions has been repeatedly emphasised (4, 41), and this study provides empirical data to support this argument. Furthermore, it also indicates the specific issues that stakeholders should discuss. Discussing with stakeholders as early as possible on how useful an AI device being developed will be in healthcare, what potential impacts and concerns it may have, and what measures should be taken to avoid them is crucial. The fundamental questions discussed in the previous section must be addressed during this process. To facilitate this, mechanisms for involving stakeholders in discussions, such as those highlighted by the AIDE project (42), will be necessary. Focus group interviews, such as those conducted in this study, can also be considered a useful method for gathering valuable insights.

### 4.3 Beyond regulating AI as a device to designing a system for an AI-based healthcare ecosystem

As mentioned in Section 1 (introduction), past regulations in various countries regarding medical AI have focused primarily on AI technologies and devices; however, discussions on how AI should be used have been insufficient. It is necessary to consider the usefulness of the developed AI devices and the entire healthcare system in which they will be implemented. In Japan, the Ministry of Health, Labour, and Welfare has clarified that the responsibility for using AI in healthcare presently lies with the doctors (7), while the Japan Medical Association and the Primary Care Association have published documents regarding the use of AI in medical practice and its considerations (8, 11). While these represent important starting points for the discussion, moving forward, discussions involving stakeholders and policymakers on the broader healthcare ecosystem based on AI technologies are essential.

Re-examining the nature of healthcare and its systems in light of innovative technologies such as AI, discussing the various real-world impacts that these AI devices will bring, and establishing practical rules for their use in healthcare settings are essential steps toward the future design of healthcare systems. To achieve this, considerations regarding resource allocation are important, and the healthcare system must be optimised for AI integration. Simultaneously, for AI devices to be used effectively, clarifying their intended purpose, developing them accordingly, and aligning their use with the intended goals is essential. This aligns with arguments we have already made in earlier work that the ethical use of AI should be discussed from a human-centred perspective – beyond the technical trustworthiness of AI algorithms – and grounded in the relationships between humans and technology (42). Building on the present findings, we therefore recommend that AI in healthcare be developed and used through processes that involve all key stakeholders, ensuring that they are both adequately informed and meaningfully included in decision-making. Although these points arise from the Japanese context studied here, we consider them relevant to the ethical use of AI in other healthcare systems.

Furthermore, as participants in this study mentioned, educating healthcare professionals, enhancing the literacy development of patients and citizens, and appropriately allocating benefits and rights associated with AI development and use are crucial elements in ensuring the effectiveness and sustainability of AI-driven healthcare (23–25). These aspects must be considered to implement AI effectively in healthcare. Developing AI devices that are “technically useful and reliable” alone is insufficient. Therefore, to make these investments in AI truly effective, clarifying the vision of the kind of healthcare we aim to achieve through AI in the future is important.

### 4.4 Limitations

This study has some limitations. First, because the discussions were anchored in predefined scenarios, participants’ responses will have been shaped by these prompts and will have focused on those specific AI applications and situations. This may limit the transferability of the findings beyond the scenarios presented.

Second, our recruitment strategy may have introduced selection bias. As recruitment relied largely on the research team's professional networks, participants from the tertiary healthcare institutions were overrepresented and, as a result, will be differently positioned to many of the changes discussed than HCPs working in the primary sector. Participation was voluntary and required availability for online group interviews, which may have favoured individuals with greater interest in AI or more flexible schedules. In addition, some of the doctors classified as “closely involved with AI” had previously engaged with earlier phases of the AIDE project, and may therefore have been more familiar with, or sensitised to, human-centred discussions about AI.

Third, the relatively small sample and the scope of professions and specialties represented may limit the transferability of the findings to the full diversity of HCPs in Japan. For example, we did not include HCPs specialising in psychiatry, paediatrics, intensive care, or palliative care, nor professionals such as physical therapists, occupational therapists, or case workers. Nevertheless, as a qualitative study, our aim was not statistical generalisation but to generate rich insights into the meanings, perceptions, and expectations that HCPs hold regarding AI in healthcare.

Fourth, the age distribution of participants was skewed towards younger and mid-career HCPs, with relatively few participants aged 50 years or older. As participation required joining an online focus group, HCPs who were less familiar or comfortable with digital tools may have been less likely to take part. As a result, perspectives that may vary by age and digital experience—particularly those of older HCPs—may be under-represented, which may limit the transferability of the findings.

Finally, the critical-reflective positioning of the researchers may have influenced interpretations. AK, who conducted the facilitation and primary analysis, has a background as a physician, which could have influenced the interpretation of the participants' statements from a doctor's perspective. However, to mitigate potential bias, BY, a non-medical professional, co-facilitated and collaborated closely with AK to carefully analyse the data.

## 5 Conclusion

This study explored the perspectives of HCPs in Japan regarding AI development, implementation, and use in healthcare, highlighting their expectations and concerns. Through focus group interviews based on scenarios, participants shared nuanced views on the potential benefits and risks of AI, and raised fundamental questions about the concepts previously taken for granted in conventional medicine, including “disease”, “the patient”, and “healthcare”.

Our findings emphasise the importance of involving stakeholders – particularly frontline clinical care professionals – in the early stages of AI development and use. Without such engagement, even technically advanced AI systems may fail to integrate effectively into real-world healthcare settings. This study also revealed the need to move beyond device-level regulations and towards the design of a broader ecosystem for AI-based healthcare, encompassing considerations such as professional responsibility, patient autonomy, resource allocation, and health equity.

While these insights provided are contextually grounded in Japan, the challenges and reflections articulated by HCPs resonate globally. As AI continues to transform healthcare, designing inclusive, anticipatory, and human-centred governance frameworks will be essential. This study provides empirical evidence supporting such efforts and demonstrates that deliberative engagement with diverse stakeholders is not only necessary but also a foundation for building trusted and sustainable AI in healthcare.

These findings suggest several implications for policy, practice, and future research. AI developers and vendors, together with clinical leaders in healthcare organisations, should consider involving frontline HCPs as well as patients and citizens at the earliest stages of design and procurement to clarify intended use, assess clinical utility, and anticipate downstream consequences (e.g., additional investigations, system burden, and potential psychological impacts associated with ultra-early detection). Health authorities and regulators, together with relevant professional organisations and specialty societies, could develop and periodically update practical guidance—co-produced with stakeholders—on responsibilities, communication and consent expectations, and procedures for situations in which AI outputs diverge from clinical judgement. Public funders may also consider requiring evidence of meaningful upstream stakeholder engagement and consideration of system-level impacts as part of funding decisions for healthcare AI. Future studies should examine how such engagement and governance arrangements operate in practice and include a wider range of specialties and care settings.

## Data Availability

The datasets presented in this article are not readily available because we have to protect participants' confidentiality. Public disclosure of the data would conflict with the ethical agreement signed by the participants and approved by the University of Osaka Hospital Ethical Review Board. However, access to certain aggregated data may be granted upon approval by the Review Committee. Requests to access the datasets should be directed to the University of Osaka Hospital Ethical Review Board at: rinri@hp-crc.med.osaka-u.ac.jp.
