# Supplementary material for: Perspectives on the use of artificial intelligence in Japan: a focus group interview study of healthcare providers
Source: Front Digit Health. 2026 Feb 11;8:1716687. doi: 10.3389/fdgth.2026.1716687 (PMC12932437; doi:10.3389/fdgth.2026.1716687)
Supplement: Supplementary file 1 [file Datasheet1.docx]

**Scenario 1: Early detection of lung cancer**

Seventy percent of lung cancers are detected in the later stages of the disease, when it is hard to treat. Leading clinical academics at several universities and hospitals are collaborating with an AI company to implement an artificial intelligence (AI) algorithm to help diagnose lung cancer earlier. This is a self-learning algorithm trained on huge amounts of human-labelled scanned images and outcomes, that can detect patterns in new data without human input.

If successful, the algorithm could be used to assess medical scans collected during routine care, or through a screening programme for at-risk individuals. It is expected that the algorithm will be able to identify early signs of cancer, even before they are visible to the human eye.

The algorithm is undergoing validation, meaning before it can be routinely used with patients, it will need to be tested and approved by the relevant regulatory agencies. If approved, the algorithm will determine how likely it is that someone has or will develop lung cancer, allowing doctors, along with patients, to make decisions about what steps should be taken. These might include taking a blood test or a biopsy (a tissue sample), or setting up a system of closer monitoring of patients identified as being at high risk – perhaps through more frequent scanning. This could allow any necessary treatment to begin as soon as possible, thus improving outcomes, and increasing life expectancy.

Vast amounts of data are required to train the algorithm. The data would include lung scan images and associated medical records from lung cancer patients, and from heathy individuals who did or did not go on to develop lung cancer. To develop and train an algorithm of this type, the company requires access to millions of scanned images. The partnering hospitals have agreed to provide the company access to 25 million lung scans. In exchange for access to data, the Trust/ partnering hospitals will be given a financial stake in the company.

Patients at participating hospitals have not given consent for their data to be used by a private company in this way. If the algorithm is successful, both company and the partnering Trust/ hospitals will profit from the algorithm, but patients and the doctors who were involved in the past cases will not be compensated for the use of this medical data in developing the algorithm.

The training dataset needs to have enough diversity for the algorithm to be good at detecting lung cancer in any patient. Currently, there are fewer scans from females in the dataset used to develop the algorithm, so the resulting algorithm may not be sufficiently trained to detect lung cancer as effectively in females as in males. Additionally, the results of the algorithm must also be interpretable by clinicians who use it to support their clinical decision-making. A key characteristic of this type of algorithm is that how it derives recommendations from its processing of data is often unclear.

**Scenario 2: Using speech recognition in doctor-patient consultations**

In large hospitals, doctors, nurses and other clinical staff, daily, need to record consultations and any procedures as clinical notes that are uploaded into the electronic patients records. On top of this, there is a huge amount of correspondences between healthcare professionals about patients and their care, and this communication can be in either paper or electronic formats. Any such correspondence must also be uploaded in to the patient’s records.

Some clinicians dictate notes and then get them transcribed and uploaded. Others, type up their notes during or even after a consultation using a computer or tablet. Some patients may feel that a doctor making notes during consultation makes it impersonal, especially if clinicians make little eye contact with patients because they are typing. If notes are created after the consultation and from memory there could be errors.

Where dictated noted needs to be transcribed, either by secretarial staff or by being outsourced to a company, the time needed to complete notes and letters can be up to two weeks. This slows down referrals and other important forms of correspondence. It is also very costly to outsource subscription service.

For all the above reasons, clinical correspondence and patient records are areas of clinical administration that have a huge scope for AI technologies to make time and cost saving efficiencies that will also benefit the patients with greater speed of correspondence and possible the reduction of mistakes. The expectation is that it will also give clinicians more quality time with patients.

Clinical researchers an B University Hospital, have been working in collaboration with a speech software company to develop an AI algorithm aimed at cutting the cost and time required for writing clinical notes and correspondence. A speech recognition system records and transcribes consultations in real time. The algorithm is being trained to learn to accurately transcribe real-time speech, including the many different accents and speech patterns of real-life clinicians and patients, and to identify medical terminology used in consultations. It is expected that the AI system will make be able to make accurate decisions about what part of the recorded consultation is relevant or not, and where to insert this into different fields of the patient record. The final command to upload the clinical notes is done by the clinician. This data will go into the electronic patient records. The technology can also be used for clinical correspondence based on dictation.

A pilot study has shown that the AI system significantly reduces workload and speeds up patient related correspondence. The clinicians in the departments using the technology, especially oncology, cardiology and pediatrics, reported a very high level of satisfaction with the system and letter turn-around time was greatly reduced. At this stage we have no data on whether patients are comfortable with consultations being recorded.

**Scenario 3: AI-assisted health monitoring**

For patients with long-term health conditions such as diabetes, it is important that they can self-manage their condition as much as possible. For example, by measuring their blood glucose level, blood pressure and/or heart rate patients with diabetes or other chronic conditions can improve their overall health.

A private technology company has collaborated with a group of public hospitals in City A to develop an AI algorithm, a set of programming rules for looking at and sorting data, to detect when a person is at risk of certain medical emergencies such as blood clots or stroke based on an irregular heart rate signal. The company has developed the algorithm using millions of data points gathered from hospital electronic records such as known patient risk factors and related clinical test results. Usually, an individual can only be identified at risk for these medical emergencies if the heart’s rhythm and electrical activity are monitored through an ECG (electrocardioagram) conducted in hospital.

Before the medical records were shared with the company, any information that would identify a patient, such as name and date of birth, was removed. All data was then deleted once the algorithm had been developed. The algorithm will run on the next generation of the company’s smartwatches. Through the smartwatch, the AI algorithm is designed to set an alert system in place that will result in an ambulance being called if it detects signs of severe problems like stroke. The aim is for an ambulance to dispatch when early indications are detected to increase survival and quality of life outcomes of patients

Questions to be asked after each scenario

1. How do you feel about AI being used for this purpose?
2. What might be the benefits of this use of AI? Who would benefit?
3. Do you have any, concerns about using AI in this situation?

General questions to be asked apart from the scenarios

1. Do you, expect your professional role to change if AI were to be adopted into your clinical practice? If yes, how? How does that make you feel?
2. Who should be involved in the development and implementation decisions for AI uses in healthcare? Are there any groups specific in your area of work that should be involved in decisions pre and post implementation of AI?
